# Quality Enhancement and In Vitro Starch Digestibility of Wheat–Yam Composite Flour Noodles via Adding Different Improvers

**DOI:** 10.3390/foods14101654

**Published:** 2025-05-08

**Authors:** Shuo Hu, Kai-Nong Sun, Qiu-Jia Peng, Run-Hui Ma, Zhi-Jing Ni, Kiran Thakur, Zhao-Jun Wei

**Affiliations:** 1 School of Biological Science and Engineering, Specialty Food Nutrition and Health Innovation Team of Ningxia Hui Autonomous Region, North Minzu University, Yinchuan 750021, Chinalovebear@vip.163.com (Z.-J.N.); kumarikiran@hfut.edu.cn (K.T.); 2 School of Food and Biological Engineering, Hefei University of Technology, Hefei 230601, China

**Keywords:** Chinese yam, TGase, improver, noodle quality, in vitro starch digestibility

## Abstract

The addition of Chinese yam powder (CYP) to wheat flour (WF) can compromise the elasticity of noodles due to weakening of the gluten network. To address this, we investigated the effects of TGase, vital wheat gluten (VWG), and egg white powder + sodium alginate (EWP + SA) on the quality of wheat yam composite flour noodles (color, cooking, textural, thermal properties, and in vitro starch digestibility). Our findings demonstrated that VWG, TGase, and EWP + SA exert distinct yet complementary effects on the quality of composite flour noodles. Combining TGase and VWG yielded the densest microstructure and better textural properties, including hardness, adhesiveness, and springiness. TGase and EWP + SA addition significantly increased slow digestible starch (SDS) content (G6: 33.81%) while reducing starch digestibility. These findings demonstrate that synergetic combinations of improvers, particularly TGase with VWG or EWP + SA, improve both the processing characteristics and nutritional quality of yam-based products.

## 1. Introduction

The Chinese yam is highly popular across China and is commonly consumed as a vegetable, as a staple meal, and as an important source of Traditional Chinese Medicine [1]. Recent experimental research has shown that, in addition to its antioxidant and anti-tumor functions, Chinese yam can lower blood sugar and blood lipid levels and improve liver damage [2]. Hence, Chinese yam can be regarded as a functional food with medicinal origin, and its nutritional value and anti-disease mechanism are gradually being explored. These aspects have become a hot spot in the latest functional food research.

Fresh yam is prone to oxidation, so the mold and rot during its storage make it difficult to achieve large scale mechanized exploitation and utilization [3]. To address this, it is usually processed into powder and added to composite flours or used in food production as an important ingredient. In our previous study, Chinese yam flour was partially substituted for wheat flour to produce functional wheat–yam composite flour noodles. It was reported that adding 30% Chinese yam flour could greatly improve the rheological characteristics and texture of composite flour noodles [4].

Although yam flour has a high nutritional value, including an adequate amount of dietary fiber and a full range of important amino acids and other minerals, it is relatively low in protein [5]. The quality of the noodles is significantly influenced by the strength of the gluten protein network formed by the proteins [6], which is essential for noodle processing and storage. When the protein content of the flour is low, the noodles are poorly processed and their structure tends to disintegrate. Poorly elastic noodles are prone to breaking, exhibit greater cooking losses, and have a poor chewy texture after steaming. To compensate for the low gluten protein content and lack of elasticity of Chinese yam flour, the quality of the noodles can be further improved by adding various improvers.

Transglutaminase (TGase) is a commonly used modifier that catalyzes acyl transfer reactions. It can cross-link protein molecules, thereby altering protein properties. Its cross-linking properties are widely used in the food industry [7]. TGase treatment can effectively restore the dough structure by cross-linking gluten proteins, increasing the strength of the gluten network, improving the elasticity and toughness, and enhancing the rheological and baking properties of the dough [8]. Yam flour is low in gluten protein, and normally gluten-free raw flour is fortified with TGase, which also boosts the nutritional value of the noodles. Proteins commonly used to enhance the protein cross-linking reaction of TGase include soy isolate, whey protein, and ovalbumin [9]. So far, TGase has shown promising results in increasing various protein properties, including emulsification, water retention, foaming, and elasticity in food [10].

Vital wheat gluten (VWG), a nutrient-rich source of vegetable protein, is a common natural flour quality improver. As a natural gluten enhancer, it has been widely used in a wide range of pasta products to strengthen the gluten network and improve dough adhesiveness, elasticity, and elongation [11]. Starch is the main component of yam–wheat composite noodles, and the addition of polysaccharides and proteins can interact specifically with starch and play an important role in the structural development of starchy dough [12]. Sodium alginate (SA) is an anionic linear polysaccharide. Modern studies have shown that SA can reduce random binding between protein molecules by changing the rheological and textural properties of proteins, resulting in a more ordered protein–polysaccharide gel network structure [13]. Liu et al. (2022) showed that the dough with SA has higher volume and elasticity, along with a more compact, homogeneous, and continuous gluten network structure [14]. Egg white powder (EWP), usually obtained through spray drying, is added to pasta products to improve chewy texture and elasticity. It has been shown that egg white protein can increase pasta firmness and reduce cooking losses [15].

For this investigation, six formulations were prepared by mixing wheat–yam composite flour noodles with TGase, VWG, EWP, and SA. The effects of TGase, VWG, EWP + SA, and their interactions on the color, microstructure, cooking characteristics, textural qualities, and in vitro starch digestibility of wheat–yam composite flour noodles were examined. Our main aim was to develop a multipurpose noodle with both culinary and medicinal qualities and to elucidate the role of TGase, VWG, EWP, and SA in the quality control of wheat–yam composite flour noodles.

## 2. Materials and Methods

### 2.1. Raw Materials

Wheat flour (Golden dragon fish) was purchased from Yihai Kerry Arawana Holdings Co., Ltd. (Shanghai, China). Dried Chinese yam was purchased from Tanan Local Wholesale Store (Jiaozuo, China) and ground to a 100-mesh sieve using a Xichu food grinder (Jinhua, China). All other chemicals used were of analytical grade.

### 2.2. Noodle Preparation

Through pre-testing, the basic noodle recipe was determined as outlined in Table 1. The dough was placed in a press with a 5 mm roll spacing and repeated 3–4 times until forming a dense, hole-free sheet. Subsequently, the dough was extruded through a 5 mm wide exit hole, and the resulting noodles were manually cut to a length of 15 cm. The raw noodles were cooked to their optimal time, stored at −20 °C for 12 h, vacuum freeze-dried, and stored in a sealed container for further use [4].

### 2.3. Color Measurement

The freshly prepared noodles were cut into 3 cm × 3 cm slices each and their color was determined using a colorimeter model SC-80C. Five random measurements were taken per slice, and eight readings were averaged to obtain the LAB values [16].

### 2.4. Microstructure Determination

The microstructure of the noodles was analyzed according to the previously reported method by Sangpring et al. (2015) [17]. Cross-sections of 0.5 mm face strips were cut, mounted on stubs, and coated with gold for 90 seconds. The cross sections of the sample were then observed under a Scanning Electron Microscope (SEMJEOL JSM-5910LV, JOEL USA Inc., Peabody, MA, USA) at 18 KV, with 250× and 500× magnification.

### 2.5. Determination of Cooking Properties

The cooking loss and water absorption rate of the freshly prepared noodles were calculated using the previous method of Lu et al. (2009) [18]. Noodle samples (15 cm) were cooked in boiling water for optimal cooking time, then immediately removed, rinsed in 200 mL of distilled water for 30 s, drained for 10 min, and weighed. The noodle soup was collected in a volumetric flask. A 50 mL aliquot was placed in a drying beaker and oven-dried at 105 °C to constant weight. The residue was weighed, and the cooking loss was calculated as a percentage of the initial noodle weight. Water absorption was expressed as a ratio of the pasta mass before and after cooking.

### 2.6. Mechanical Analysis

The mechanical properties of the cooked noodle strands were assessed using the method described by Cai et al. (2023) [19] with slight modifications. Each cooked noodle strand was folded twice and the P/36R model probe was employed for the measurements. The experimental parameters were set as follows: a pre-measurement speed of 10 mm/s, a test speed of 1 mm/s, a post-measurement speed of 10 mm/s, a test time of 5 s, a compression level of 90%, and a trigger point of 10 g. For the tensile property testing, the A/KIE probe was selected. The experimental parameters for this test were set at a pre-measurement speed of 10 mm/s, a test speed of 1 mm/s, a post-measurement speed of 10 mm/s, a stretching distance of 12 mm, and a trigger point of 10 g. Each sample was tested three times to ensure reproducibility of the results.

### 2.7. Thermal Properties

Thermal characterization of the samples was performed using differential scanning calorimetry (DSC), following the method described by Zou et al. (2021) [20]. For this, the sample (5 mg) was weighed, mixed with water (1:2, *w*/*w*), and equilibrated at 4 °C in the refrigerator for 24 h. The DSC analysis was conducted over a temperature range of 20 °C to 100 °C at a heating rate of 10 °C/min, the carrier gas was nitrogen at a flow rate of 50 mL/min, and an empty crucible was used as a blank control.

### 2.8. In Vitro Digestive Properties

According to the method described by Lu et al. (2016) [21], with minor modifications, the cooked pasta samples were freeze-dried, powdered, and then sieved through 100-mesh sieves. In vitro digestion experiments were performed on the composite flour pasta, which has been supplemented with different improvers. The following formulas were used to determine DS (digestible starch), RDS (rapid digestible starch), SDS (slow digestible starch), and RS (resistant starch).

(1) $$\mathrm{DS}=0.9\times \mathrm{GG}\times 180\times \frac{V}{W}\times \left(100-M\right)$$

(2) $$\mathrm{RDS}\left(\%\right)=\left(G_{20}-G_{0}\right)\times 0.9\mathrm{TS}\times 100\%$$

(3) $$\mathrm{SDS}\left(\%\right)=\left(G_{120}-G_{20}\right)\times 0.9\mathrm{TS}\times 100\%$$

(4) $$\mathrm{RS}\left(\%\right)=100\%-\mathrm{RDS}-\mathrm{SDS}$$

GG is the glucose concentration, V is the volume of digestion solution, W is the sample mass, S is the starch content of the sample, M is the moisture content of the sample, G_20_ is the mass of glucose in the solution at 20 min of digestion, G_0_ is the mass of free glucose in the solution before digestion, G_120_ is the mass of glucose in the solution at 120 min of digestion, and TS is the mass of total starch in the sample.

### 2.9. Statistical Analysis

All data were averaged after being conducted in triplicates and analyzed using SPSS software (version 25.0) at a significant level of *p* < 0.05. Results are expressed as mean ± SD. Data were plotted using Origin 2018. For interventionary studies involving animals or humans, and other studies that require ethical approval, the approving authority and ethical approval code are listed.

## 3. Results

### 3.1. Effect on Noodle Color

**Figure 1:**
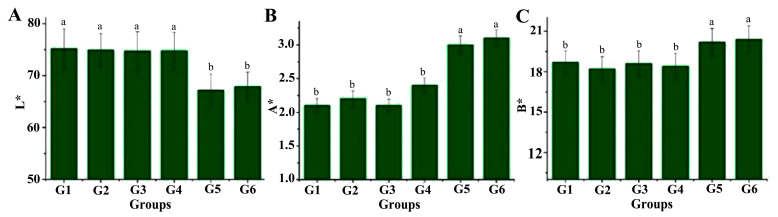
Color parameters of wheat–yam composite noodles: (**A**) L*, (**B**) A*, and (**C**) B*. Different letters (a, b) represent significant differences at *p* < 0.05 level.

Color has consistently been an important quality characteristic of pasta, as it aligns with consumer preference. Consumers generally favor noodles that retain a vibrant color, while dull grey hues are less acceptable to consumers [22]. The color parameters (L*, a*, b*) of the six types of noodles are shown in Figure 1. Notably, the addition of vital wheat gluten VWG (G2), TGase (G3), and TGase-treated gluten (G4) did not lead to significant changes in color of the noodles. Conversely, the addition of EWP + SA (G5 and G6) induced significant changes in the noodle color. Specifically, the brightness (L* value) of these noodles was significantly lower than those of the other noodle types, while the redness (A* value) and yellowness (B* value) were significantly higher. This phenomenon can likely be attributed to the reaction between the egg white powder and sodium alginate during the high-temperature processing of the noodles, which results in a marked increase in the redness (a* value) and yellowness (b* value). Additionally, the color of the noodles after the addition of TGase (G3) was not significantly different from that without TGase (G1), which coincides with the results of Yeoh et al. (2014) [23].

### 3.2. Microstructure Analysis

**Figure 2:**
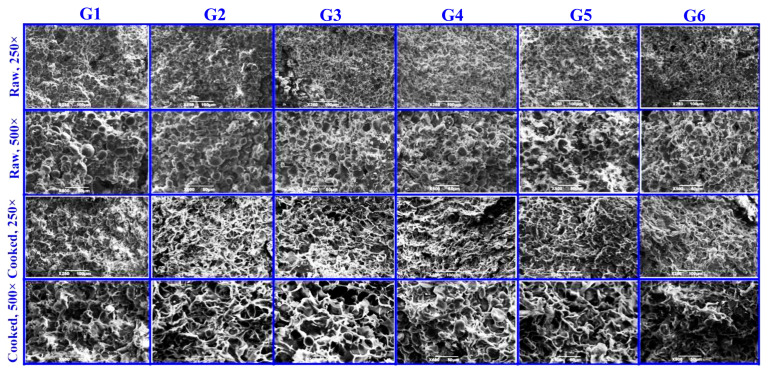
Scanning electron micrographs of wheat–yam composite noodles (cooked and raw).

The microstructure of steamed noodles is characterized by the size of the holes within the gluten protein network and the degree of starch loss during noodle heating [24]. At the same time, it allows for the visualization of interaction between the gluten protein network structure and starch. Figure 2 depicts the microstructure of the six formulations of wheat–yam composite flour noodles. It is clearly seen that the protein molecules interact and cross-link, enveloping starch granules to form a gluten protein network structure. Compared to G1, the microstructure of the noodles with the addition of VWG (G2) was flatter and more compact. With the addition of TGase (G3), the structure of the noodles was slightly uneven and the starch granules were not tightly packed. However, with the addition of TGase and VWG (G4), the microstructure of the noodles was the most dense and flat. The starch particles were tightly packed in the protein network and the gluten protein network structure exhibited the highest level of elasticity and toughness.

After steaming, noodles with a single addition of VWG (G2) and TGase (G3) exhibited larger holes in the gluten network and a loose structure. This phenomenon can likely be attributed to the addition of protein, which disperses the water bound to the starch, accelerating starch loss and, consequently, resulting in a loose structure with larger cavities. In contrast, noodles with both TGase and VWG added (G4) showed smaller holes in the gluten protein network after steaming. They retained more starch particles and featured a more intact gluten network structure, thereby better preserving the noodle microstructure.

The addition of EWP + SA (G5) formed a polysaccharide and protein co-preparation system. After steaming, the protein aggregates were interlinked, the pores of the gel microstructure became smaller, and the noodle structure was tightly bound [25]. With the addition of EWP + SA and TGase (G6), the microstructure of the noodles was flatter than G5 and the starch granules were more tightly cross-linked with the protein. These findings were consistent with the results regarding the cooking characteristics of the noodles and the textural properties determined by TPA. Egg white powder and sodium alginate are rich in free sulfhydryl groups, which crosslink with gluten proteins. The cavities in the noodles with the addition of EWP + SA (G5) expanded after steaming. This might be because sodium alginate and egg white powder form a gel during the heating process, increasing noodle stickiness and reducing starch loss [12].

### 3.3. Cooking Properties

**Figure 3:**
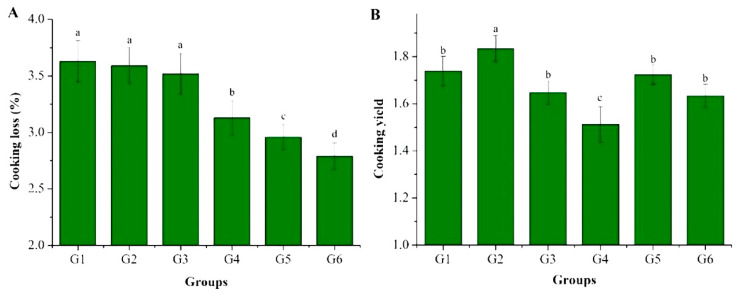
Cooking characteristics of wheat–yam composite noodles: (**A**) cooking loss; (**B**) cooking yield. Different letters (a, b, c, d) represent significant differences at *p* < 0.05 level.

For composite noodles, cooking loss and swelling are crucial quality indicators. A lower cooking loss implies less quality degradation of the pasta and higher nutrient retention. Higher swelling, on the other hand, reflects more mass gain after cooking and better commercial value. The cooking characteristics of the six formulations of wheat–yam composite noodles are shown in Figure 3. When comparing the cooking loss values of G2 and G1 and G4 and G3, it is evident that the addition of VWG decreased the cooking loss values in the noodles (Figure 3A). Moreover, the water absorption of the noodles in G2 was significantly higher compared to G1 (Figure 3B). This can be attributed to the increased protein content in the noodles with the addition of VWG, which promotes the formation of a gluten network. Consequently, this led to lower cooking losses and thus increased water absorption. However, the water absorption of G4 was significantly lower than that of G3. The addition of TGase reduced the availability of free starch by cross-linking protein molecules, embedding starch granules between them. As a result, during the heating process, water penetration into the interior of the noodles was inhibited, thereby decreasing the water absorption of the noodles. In this case, the impact of TGase on reducing water absorption outweighed the water absorbing effect of VWG.

A comparison of the cooking loss and water absorption of G3 and G1, G4 and G2, and G6 and G5 noodles showed that the addition of TGase significantly reduced the cooking loss and water absorption of the noodles. With the addition of TGase, cross-linking between proteins was improved, resulting in a more stable gluten network and a significant reduction in cooking losses. At the same time, the network structure showed a denser and elastic nature by cross-linking between protein molecules, thus reducing the swelling of the noodles.

The comparison between G5 and G1 and G6 and G3 showed that the cooking loss of the noodles decreased significantly after the addition of EWP + SA but the water absorption did not change much. This is consistent with the results of Hong et al. (2021) [26] stating that SA improved noodle quality and reduced cooking losses and water absorption regardless of SA concentration. The probable reason might be that the egg white protein is cross-linked with gluten proteins by free sulfhydryl groups during heating, which gelatinizes and wraps the starch granules, while sodium alginate can wrap the starch granules by microencapsulation, preventing the dissolution of starch on the surface of the pasta during cooking.

### 3.4. Mechanical Properties

Mechanical properties are among the most crucial characteristics for evaluating the quality of pasta products and determining consumer acceptance. As shown in Table 2, when compared to G1, the addition of VWG increased the hardness and decreased the elasticity of the noodles (G2). This is likely because the addition of VWG enhanced the strength of the protein network structure, thereby increasing the hardness of the noodles. During the cooking process, protein gelation takes place, and the increased protein concentration compacts the gel network, thus reducing the elasticity of the cooked pasta. After cooking, the hardness, elasticity, adhesiveness, and cohesiveness of the noodles with both VWG and TGase (G4) were significantly improved compared to those with VWG alone (G2). The above results indicate that the noodles treated with TGase after the addition of VWG can better cross-link with endogenous wheat proteins. This cross-linking significantly improved the elasticity and viscosity of the noodles, reduced the loss of starch granules during cooking, and thus, improved the textural properties of the cooked noodles. Weng et al. (2020) reported that noodles with 2% TGase addition exhibited higher values for hardness, springiness, cohesiveness, gumminess, chewiness, and strength [27].

Compared to G1, the hardness, elasticity, and cohesiveness of the cooked noodles (G5) were reduced after the addition of EWP + SA. Compared to G5, G6 showed a significant decrease in hardness and a significant increase in adhesion and elasticity, with no significant change in cohesion. The above results suggest that noodles with the addition of EWP and SA, when treated with TGase, can facilitate the formation of protein gels and protein cross-linking. This process also enhances the elasticity and toughness of the gluten network [28] and improves the textural properties of the noodles.

### 3.5. Thermal Analysis

**Figure 4:**
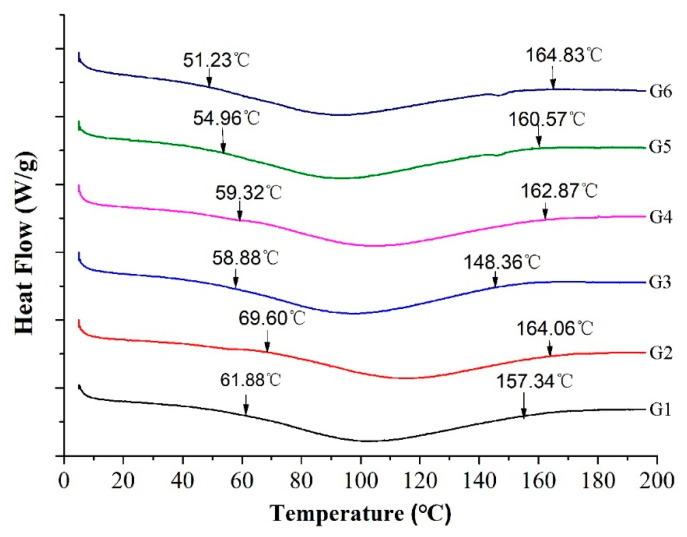
Thermal characteristics of wheat–yam composite noodles.

To further reveal the edible quality of improvers in yam–wheat composite noodles, the thermal properties of the noodles were determined for six different formulations. DSC results and parameter characterization are shown in Figure 4 and Table 3. Specific characteristic parameters included the starting pasting temperature (To), the peak pasting temperature (Tp) as the peak of the heat absorption heat flow curve, and the ending pasting temperature (Tc).

The enthalpy of pasting can be used as an indicator to evaluate the physicochemical properties of starch such as crystallinity. The simultaneous addition of VWG and TGase (G4) clearly showed a slight decrease in the To and almost no change in the Tp, but a significant increase was reported in the pasting range, the gelatinization, and the enthalpies. It is suggested that the simultaneous addition of VWG and TGase may have increased the protein content of the noodles and TGase better promoted cross-linking between the proteins, resulting in a more stable protein network structure [29].

Compared to G1, G5 had a significantly lower To and Tp, but a significantly higher pasting range and gelatinization enthalpies. Compared to G5, G6 also had lower To and Tp, but a significantly higher gelling range and gelatinization enthalpies. The above results revealed that the addition of EWP + SA introduces a polysaccharide component in the protein network structure to support the structure for better overall structure stability. The addition of polysaccharides leads to a significant increase in the enthalpy of pasting in the pasting range. On the one hand, the addition of EWP supplements the protein content of the noodles, and on the other hand, the EWP has a high content of sulfhydryl groups. As the noodles were heated during processing, more cross-linked disulphide bonds were formed between the egg white powder and the gluten protein, resulting in a more stable protein structure and improved heat resistance.

### 3.6. In Vitro Starch Digestibility

**Figure 5:**
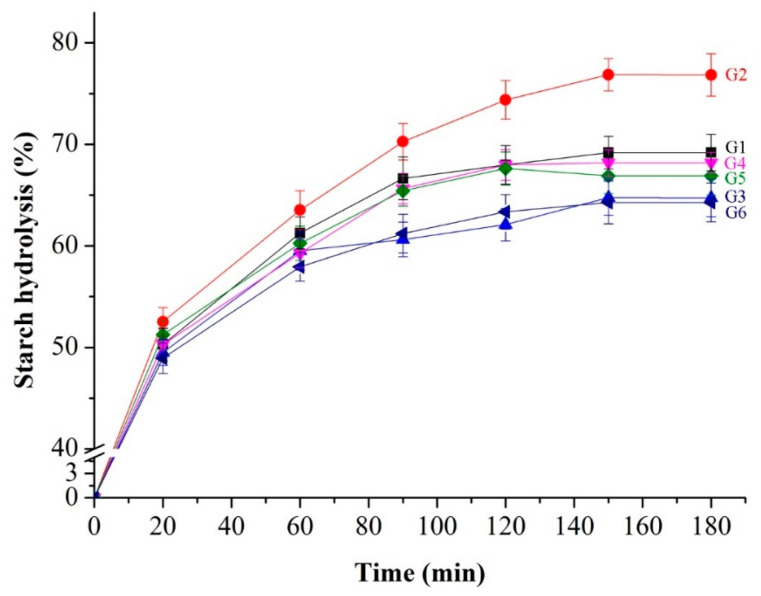
Differently simulated in vitro digestion dynamic curves of wheat–yam composite noodle starch.

Resistant starch (RS) is a key indicator of the nutritional and functional properties of starch. It is classified as dietary fiber due to its ability to partially ferment in colon. Based on its digestibility, RS can be further classified into slowly digested starch (SDS) and rapidly digested starch (RDS). The external starch digestion kinetic curve is shown in Figure 5 and the in vitro starch digestibility is presented in Table 4. Notably, the starch hydrolysis rate was significantly higher in G2 than in the other groups. When compared to the control group, the RDS content increased significantly from 55.21% to 57.70%, and the SDS content increased from 24.32% to 26.02%. These results indicate that the addition of VWG improved the digestibility of starch. This phenomenon can likely be attributed to the addition of VWG, which increased the protein content. The competition between protein molecules and starch for water then occurs, causing the starch granules in the gluten network to be displaced. This displacement facilitates the interaction between the substrate and the enzyme, thereby improving the overall digestibility.

However, the digestibility of G4 starch was even lower than that of G1 after the addition of both VWG and TGase. This suggests that TGase catalyzes the cross-linking of VWG, forming a mesh structure that traps the starch particles in the noodles, thereby reducing their digestibility. At the same time, gluten itself cannot be digested, thus contributing to the overall low level of digestibility of the noodles [30].

A comparison between G5 and G1, as well as G6 and G3, shows that the addition of the EWP + SA improver significantly reduced digestibility and increased SDS content. Sodium alginate, a common dietary fiber, has indigestible properties. It acts as a physical barrier between the starch granules and the digestive enzymes, reducing the extent of starch hydrolysis through microencapsulation. Simultaneously, sodium alginate readily binds with water and competes with starch for water molecules. This competition diminishes the catalytic activity of digestive enzymes and slows down the rate of starch hydrolysis, aligning with the findings of Jang et al. (2015) [31].

When comparing the digestibility of starch in G3 and G1 and G4 and G2, as well as G6 and G5, it became evident that the digestibility of starch in the G3, G4, and G6 groups was lower than that in the corresponding G1, G2, and G5 (Table 4). This indicates that the pasta treated with TGase enzyme showed significantly reduced starch digestibility. TGase catalyzed the cross-linking of proteins, forming a strong mesh structure that confined the starch granules within the pasta. By restricting the movement of the substrate and the action of the enzymes, this structure effectively decreased the digestibility of the pasta.

## 4. Conclusions

Our findings illustrated that wheat–yam composite noodle flour treated with TGase and supplemented with VWG and EWP + SA improvers can significantly improve the structural and nutritional qualities of noodles. VWG treatment alone effectively improved starch digestibility, reduced cooking loss, increased water absorption, and improved hardness by strengthening gluten network formation, resulting in a denser and flatter structure. However, VWG treatment decreased elasticity, indicating the need to balance hardness and springiness in noodle formulation.

TGase treatment alone showed a limited structural improvement. It significantly reduced cooking loss and starch digestibility, which may be beneficial for developing low-glycemic index (GI) noodle products. However, it also decreased hardness and elasticity, leading to a slightly uneven structure with poorly packed starch granules, indicating further optimization is needed. Combining TGase and VWG significantly improved multiple quality parameters, achieving the best microstructure, better textural quality, and enhanced starch digestibility, indicating that the TGase–VWG combination is optimal for both cooking and nutritional performance of wheat–yam composite noodles.

EWP + SA treatment introduced a unique polysaccharide–protein interaction, improving thermal stability and reducing rapid starch hydrolysis, which is potentially beneficial for controlled energy release. Moreover, simultaneous addition of TGase with EWP + SA further improved slow-digesting starch, with the SDS content reaching up to 33.81%. This highlights its potential for functional food applications aimed at glycemic control. Overall, our research offers a feasible and beneficial strategy for producing high-quality functional noodles.

## Figures and Tables

**Table 1:** TGase-treated wheat–yam composite noodle ration.

Groups	YP (g)	WF (g)	VWG (g)	TGase (g)	EWP (g)	SA (g)
G1	30	70	/	/	/	/
G2	30	70	10	/	/	/
G3	30	70	/	3	/	/
G4	30	70	10	3	/	/
G5	30	70	/	/	10	3
G6	30	70	/	3	10	3

**Table 2:** Mechanical properties of wheat–yam cooked noodles.

Groups	Hardness (N)	Adhesiveness (g)	Springiness (g)	Cohesiveness (g)
G1	11,199 ± 32.6 ^a^	1276.69 ± 8.5 ^ab^	0.388 ± 0.041 ^a^	0.39 ± 0.029 ^a^
G2	12,141.9 ± 27.8 ^b^	1358.57 ± 30.2 ^b^	0.250 ± 0.036 ^b^	0.386 ± 0.061 ^a^
G3	11,801.2 ± 20.9 ^ab^	1105.38 ± 3.5 ^c^	0.339 ± 0.034 ^a^	0.338 ± 0.052 ^b^
G4	13,696.9 ± 39.1 ^c^	1550.88 ± 14.3 ^d^	0.391 ± 0.019 ^a^	0.390 ± 0.051 ^a^
G5	10,122.4 ± 36.1 ^d^	1140.76 ± 28.9 ^ca^	0.251 ± 0.046 ^b^	0.321 ± 0.029 ^c^
G6	8993.6 ± 20.7 ^e^	1237.27 ± 8.2 ^a^	0.417 ± 0.038 ^c^	0.332 ± 0.039 ^b^

The superscript letters indicate significant difference (*p* < 0.05).

**Table 3:** Thermal properties of wheat–yam composite noodles determined by DSC.

Groups	To (°C)	Tp (°C)	Tc (°C)	Tc − To (°C)	ΔH (J/g)
G1	61.88 ± 0.18 ^b^	101.36 ± 0.48 ^b^	157.34 ± 0.68 ^b^	95.46 ± 0.34 ^c^	132.9 ± 0.64 ^d^
G2	69.60 ± 0.34 ^a^	116.69 ± 0.57 ^a^	164.06 ± 0.57 ^a^	94.46 ± 0.61 ^c^	137.4 ± 0.62 ^d^
G3	58.88 ± 0.51 ^bc^	98.25 ± 0.61 ^b^	148.36 ± 0.87 ^c^	89.48 ± 0.27 ^d^	153.3 ± 0.78 ^c^
G4	59.32 ± 0.24 ^b^	103.49 ± 0.38 ^b^	162.87 ± 0.64 ^a^	103.55 ± 0.36 ^b^	157.6 ± 0.53 ^bc^
G5	54.96 ± 0.39 ^c^	92.72 ± 0.41 ^c^	160.57 ± 0.78 ^ab^	105.61 ± 0.51 ^b^	162.6 ± 0.58 ^b^
G6	51.23 ± 0.42 ^d^	91.60 ± 0.63 ^c^	164.83 ± 0.61 ^a^	113.6 ± 0.42 ^a^	179.0 ± 0.42 ^a^

To = onset temperature; Tp = peak temperature; Tc = conclusion temperature; Tc − To = gelatinization range; ΔH = enthalpy of gelatinization. The superscript letters indicate significant difference (*p <* 0.05).

**Table 4:** Starch nutritional fraction of wheat–yam composite noodles.

Groups	RDS	SDS	RS
G1	55.21 ± 0.42 ^b^	24.32 ± 0.32 ^bc^	20.47 ± 0.15 ^b^
G2	57.70 ± 0.36 ^a^	26.02 ± 0.27 ^b^	16.28 ± 0.27 ^c^
G3	54.36 ± 0.34 ^b^	17.28 ± 0.21 ^d^	28.35 ± 0.29 ^a^
G4	52.13 ± 0.24 ^bc^	23.42 ± 0.19 ^c^	21.28 ± 0.27 ^b^
G5	47.28 ± 0.49 ^c^	31.49 ± 0.37 ^a^	21.23 ± 0.37 ^b^
G6	45.73 ± 0.51 ^c^	33.81 ± 0.34 ^a^	20.46 ± 0.43 ^b^

RDS = rapidly digestible starch; SDS = slowly digestible starch; RS = resistant starch. The superscript letters indicate significant difference (*p* < 0.05).

## Data Availability

The original contributions presented in the study are included in the article. Further inquiries can be directed to the corresponding author.
